# PATIENT PORTAL USE WITHIN OLDER ADULTS' FAMILY CAREGIVING NETWORKS

**DOI:** 10.1093/geroni/igac059.1476

**Published:** 2022-12-20

**Authors:** Julia Burgdorf, Chanee Fabius, Jennifer Wolff

**Affiliations:** Center for Home Care Policy & Research, VNS Health, New York, New York, United States; Johns Hopkins Bloomberg School of Public Health, Baltimore, Maryland, United States; Johns Hopkins Bloomberg School of Public Health, Baltimore, Maryland, United States

## Abstract

The patient portal (hereafter, “portal”) is an online platform allowing patients to view health information, perform health management tasks, and directly message clinicians. Millions of older adults co-manage their health care with family caregivers, yet little is known about the extent to which caregivers use the portal alongside or on behalf of patients. We examined linked data from the 2017 National Health and Aging Trends Study (NHATS), National Study of Caregiving (NSOC), and American Community Survey for 1,675 (weighted n=8,511,568) older adults with disability. We measure older adult and caregivers’ respective use of the older adult’s portal from NHATS and NSOC. We use weighted, multivariable, ordered logistic regression to model transition from older adult using the portal alone, to co-using the portal with caregivers, to a caregiver using the portal on the older adult’s behalf. We find that 9% of older adults use the portal on their own, 2% co-use the portal with a caregiver, and 15% have a caregiver who uses the portal on their behalf (74% have no portal use). In adjusted models, older adults had higher odds of a caregiver using the portal on their behalf (compared to the older adult using the portal alone or in tandem with a caregiver) if they were older (aOR: 1.12; p< 0.001), female (aOR: 2.08; p< 0.01), had probable dementia (aOR: 7.04; p< 0.001), were homebound (aOR: 3.78; p< 0.01), attended doctors’ visits with a caregiver (aOR: 3.10; p< 0.01), or lived in a census tract with low broadband accessibility (aOR: 5.76; p< 0.01).

